# The impact of sleeping with reduced glycogen stores on immunity and sleep in triathletes

**DOI:** 10.1007/s00421-016-3446-3

**Published:** 2016-08-04

**Authors:** Julien Louis, Laurie-Anne Marquet, Eve Tiollier, Stéphane Bermon, Christophe Hausswirth, Jeanick Brisswalter

**Affiliations:** 1 Research Institute for Sport and Exercise Sciences, Liverpool John Moores University, Byrom Street, Liverpool, L3 3AF UK; 2 Laboratory of Sport, Expertise and Performance, French National Institute of Sport, Expertise and Performance, Paris, France; 3 Laboratory of Human Motricity, Education, Sport and Health, University of Nice Sophia-Antipolis, Nice, France; 4 Institut Monégasque de Médecine et Chirurgie du Sport, Monte Carlo, Monaco

**Keywords:** Carbohydrate, Dietary manipulation, Immune response, Sleep pattern, Endurance training, Upper respiratory tract infection

## Abstract

**Purpose:**

We investigated the effects of a 3-week dietary periodization on immunity and sleep in triathletes.

**Methods:**

21 triathletes were divided into two groups with different nutritional guidelines during a 3-week endurance training program including nine twice a day sessions with lowered (SL group) or maintained (CON group) glycogen availability during the overnight recovery period. In addition to performance tests, sleep was monitored every night. Systemic and mucosal immune parameters as well as the incidence of URTI were monitored every week of the training/nutrition protocol. Two-ways ANOVA and effect sizes were used to examine differences in dependent variables between groups at each time point.

**Results:**

The SL group significantly improved 10 km running performance (−1 min 13 s, *P* < 0.01, *d* = 0.38), whereas no improvement was recorded in the CON group (−2 s, NS). No significant changes in white blood cells counts, plasma cortisol and IL-6 were recorded over the protocol in both groups. The vitamin D status decreased in similar proportions between groups, whereas salivary IgA decreased in the SL group only (*P* < 0.05, *d* = 0.23). The incidence of URTI was not altered in both groups. All participants in both groups went to bed earlier during the training program (SL −20 min, CON −27 min, *P* < 0.05, *d* = 0.28). In the SL group, only sleep efficiency slightly decreased by 1.1 % (*P* < 0.05, *d* = 0.25) and the fragmentation index tended to increase at the end of the protocol (*P* = 0.06).

**Conclusion:**

Sleeping and training the next morning regularly with reduced glycogen availability has minimal effects on selected markers of immunity, the incidence of URTI and sleeping patterns in trained athletes.

## Introduction

Training strategies with low glycogen availability are increasingly used by endurance athletes in an attempt to improve performance thus also increasing scientific interest for exercise physiologists (Bartlett et al. 2015). The main expected effect of “training low” is to enhance training stress and thus physiological adaptations related to endurance performance. Several studies have reported an enhanced expression of a number of genes related to the stress response, substrate utilization, and mitochondrial biogenesis in athletes experiencing training low strategies compared to training with normal glycogen availability (Bartlett et al. 2015; Impey et al. 2016). Several “training low” strategies are used by endurance athletes such as training in a fasted state (i.e., 6–10 h after the last meal), training twice per day (where the second session is performed with reduced glycogen stores), or restricting carbohydrates (CHO) intake during the recovery period after exercise (Hansen et al. 2005; Morton et al. 2009; Van Proeyen et al. 2011; Yeo et al. 2008). Recently, the “sleeping low” strategy has been introduced, consisting of training in the evening followed by overnight fast and performing a subsequent training session in the morning, to accentuate the glycogen deprivation without altering exercise intensity (Lane et al. 2015; Marquet et al. 2016). Taken together, results from the literature suggest that the most stressful training situation may provide the greatest physiological adaptation. However, it is well known that increasing training stress could influence immune function and increase the risk of illness and/or injury limiting improvements in performance (Gleeson 2007).

In endurance sports, training load, nutrient intake and sleep are key factors modulating immune function either positively or negatively (Gleeson 2007; Nieman 2000). Regular physical activity, as well as balanced diet and enough sleep are associated with improved immune function (Fullagar et al. 2015; Nieman 1998). On the contrary, prolonged or strenuous exercise, energetic deficit and a lack of sleep, decrease immune function and increase susceptibility to infections and pathologies. Several studies have reported an increased prevalence of upper respiratory tract infections (URTI) during overload training programs and after endurance events such as marathon races (Bermon 2007; Pyne et al. 1995). For example, Nieman et al. (1990) recorded that 13 % of participants reported URTI during the week following the Los Angeles Marathon race and 40 % reported at least one episode of URTI during the 2 months prior to the race. Many components of the immune system exhibit changes after endurance exercise (Hoffman-Goetz and Pedersen 1994). This alteration in immune function reaches its summit within a window of 3–72 h after exercise reflecting the physiological stress the endurance athlete’s body is experiencing (Gleeson 2007). During this “open window” phenomenon, the body’s ability to fight infections is dramatically lowered, typically associated with changes in leukocyte counts and production of interleukins and immunoglobulins (Gleeson 2007). A reduction in carbohydrate availability during exercise may accentuate the alteration of immune function. Several authors have shown that very low CHO diets (<10 % of energy intake from CHO) induce an alteration of immune response compared to normal or high CHO diets (Bishop et al. 2001a; Mitchell et al. 1998). Although normal or high CHO availability during endurance exercise is effective in attenuating some immune perturbations, there is still no evidence that these beneficial effects on immune parameters are clinically relevant.

In addition to adapted dietary intake, optimized sleep patterns constitute a prerequisite for a good assimilation of training load (Myllymaki et al. 2011; Samuels 2008). Sufficient sleep participates greatly in the maintenance of the body homeostasis necessary to endure training sessions apart from injuries and infections. More than the sleep duration, the rhythmic cycle of sleep and wakefulness have important implications on the regulation of several hormones involved in the management of fatigue and immune function (Shepard and Shek 1996). Although regular exercise has been shown to improve sleep quality, the latter can be affected during intense training periods (Hausswirth et al. 2014; Leeder et al. 2012; Taylor et al. 1997). A significant reduction in sleep duration (−6 %), sleep efficiency (−2 %) and time in bed (−3 %), and an increase in wakefulness after sleep onset (+3 %), were recorded in classical ballet dancers at the end of 67 days of high physical training before a premiere performance (Fietze et al. 2009). Similarly, Hausswirth et al. (2014) reported a significant decrease in sleep duration (−7.9 %), sleep efficiency (−1.6 %) and immobile time (−7.6 %) after 3 weeks of overload training in triathletes. These sleep disturbances were associated with a higher prevalence of URTI (+67 %) compared with a control group without increase in training load. Additionally, some studies suggest that nutritional feedings may alter the sleep quality (Halson 2014). Consuming CHO in a solid meal could reduce sleep latency, while diets rich in protein may result in enhanced sleep quality by limiting wake episodes (Afaghi et al. 2008; Lindseth et al. 2013). There is limited research in this area and additional studies are necessary to evaluate the influence of habitual diet on sleep patterns, and the combined effects of strenuous exercise and nutritional manipulation.

The aim of this study was to assess whether the sleep low strategy, consisting of sleeping with reduced glycogen availability might alter sleep patterns (i.e., sleep quantity and quality) and immune response in trained triathletes. Using the same experimental protocol, this study is a complement to that presented by Marquet et al. (2016) giving us the opportunity to provide new insight on the potential side effects of the promising sleep low strategy. In light of past literature, we hypothesized that when sleeping low, athletes would experience an alteration of immune function accompanied with a higher prevalence of URTI, as well as disturbances in sleep patterns.

## Methods

### Participants

Twenty-one trained male triathletes volunteered to participate in this study. Subjects could be included if they were currently healthy, aged 18–40 years, had been involved in endurance training and competition for at least 2 years, and trained at least 10 h per week including several moderate to high intensity training sessions per week. Their mean (±SD) age, height, body mass, maximal oxygen uptake, and maximal aerobic power were 31 ± 4.7 years, 1.79 ± 0.05 m, 71.6 ± 4.5 kg, 4.2 ± 0.4 L.min^−1^, 336.6 ± 31.4 W. Before the experiment a cardiologist examined all the participants to check they did not present contraindications to physical exercise and to ensure normal electrocardiograph patterns. All subjects were free of URTI symptoms for at least 2 weeks and had not taken any medication in the 4 weeks prior to the study. The experimental design of the study was approved by the local Ethics Committee (Paris IDF VI, France) and was carried out according to the Declaration of Helsinki. After comprehensive verbal and written explanations of the study, all subjects gave their written informed consent to participate.

### Study design

**Fig. 1:**
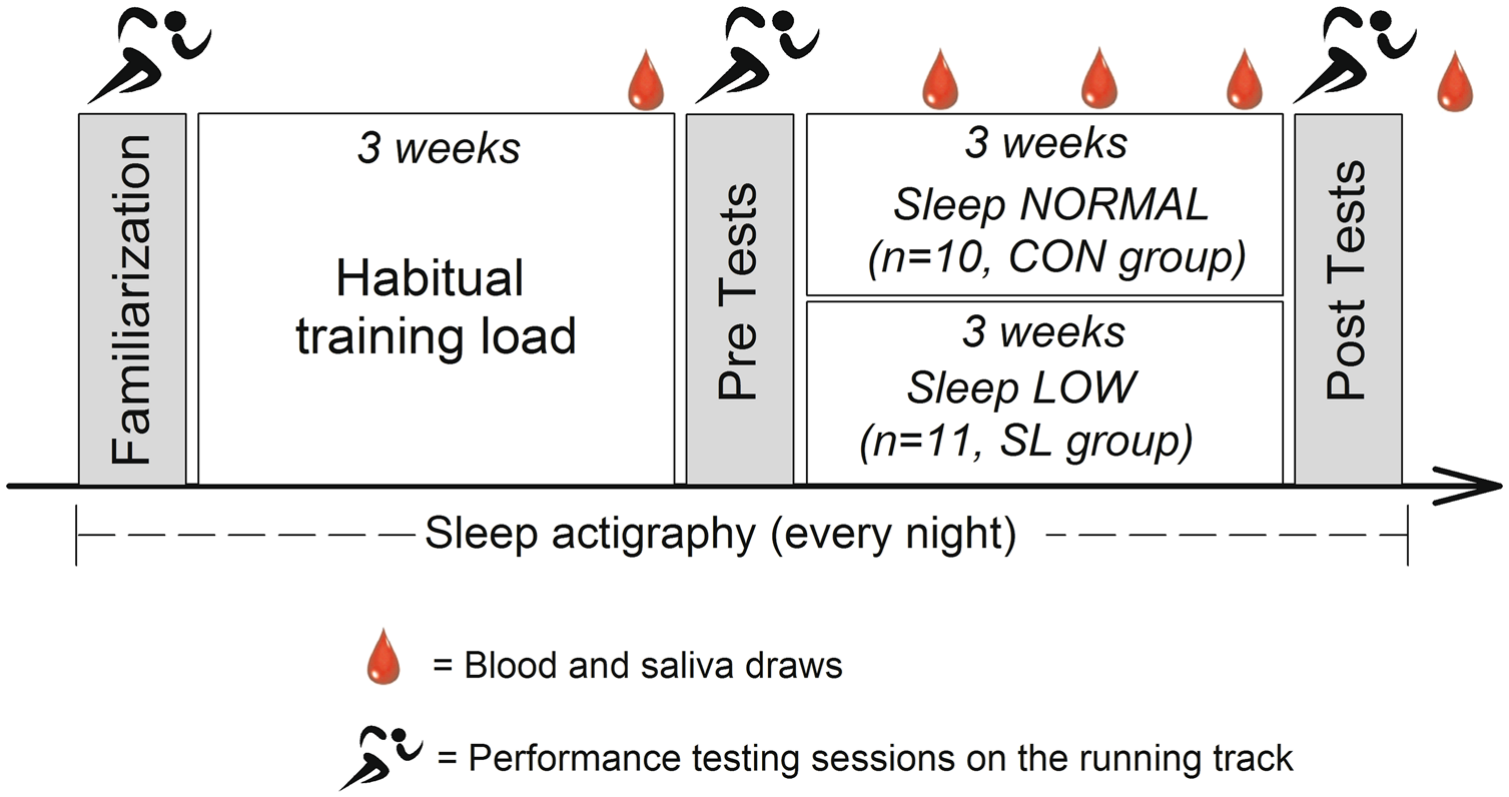
Schematic representation of the experimental protocol

An overview of the study design is shown in Fig. 1. This study was conducted to analyze the potential effects of chronic reduced glycogen availability during and between endurance training sessions on sleep pattern, selected immune parameters and the incidence of URTI. 21 trained triathletes were randomly assigned to two different groups undertaking the same endurance training program for three consecutive weeks with different nutritional guidelines. Although one group was instructed to reduce its CHO intake during and between training sessions according to a sleep low design (named Sleep Low group, SL group), the other group maintained regular CHO intake over the day (named Control group, CON group) so that the SL group slept with low glycogen availability while the other group slept with normal CHO availability. In addition to performance tests in running, sleep patterns, immune parameters and the incidence of URTI were regularly assessed before, during and after the training program.

### Training protocol

After preliminary testing sessions, all participants were involved in a 6-week training program, which was divided into two distinct phases interspersed with testing sessions. The first phase (I) consisted of 3 weeks during which the participants completed their usual training regime (10–15 h per week at various intensities). This first training period was organized to ensure that all participants were regularly involved in an endurance training and in a similar training status before the beginning of the study. The second phase (II) consisted of 3 weeks during which all participants completed the same standardized training program with different nutritional guidelines. The training program consisted of six training sessions over four consecutive days, including high intensity training (HIT) sessions in the afternoon (after 5 pm) and low intensity training (LIT) sessions in the next morning (before 10 am). The training intensity was individually set according to the individual maximal aerobic power (MAP). All LIT sessions consisted of 60 min cycling at 65 % MAP, while HIT sessions consisted alternatively of 8x5 min cycling at 85 % MAP and 6x5 min running at individual 10 km intensity with 1 min recovery between sets. One LIT session per day was prescribed for the other days of the week to maintain a 10–15 h training volume. All participants performed all the training sessions in their own training structure and were monitored (activity, duration, intensity, rate of perceived exertion) and controlled by heart rate recordings.

### Nutritional protocol

**Fig. 2:**
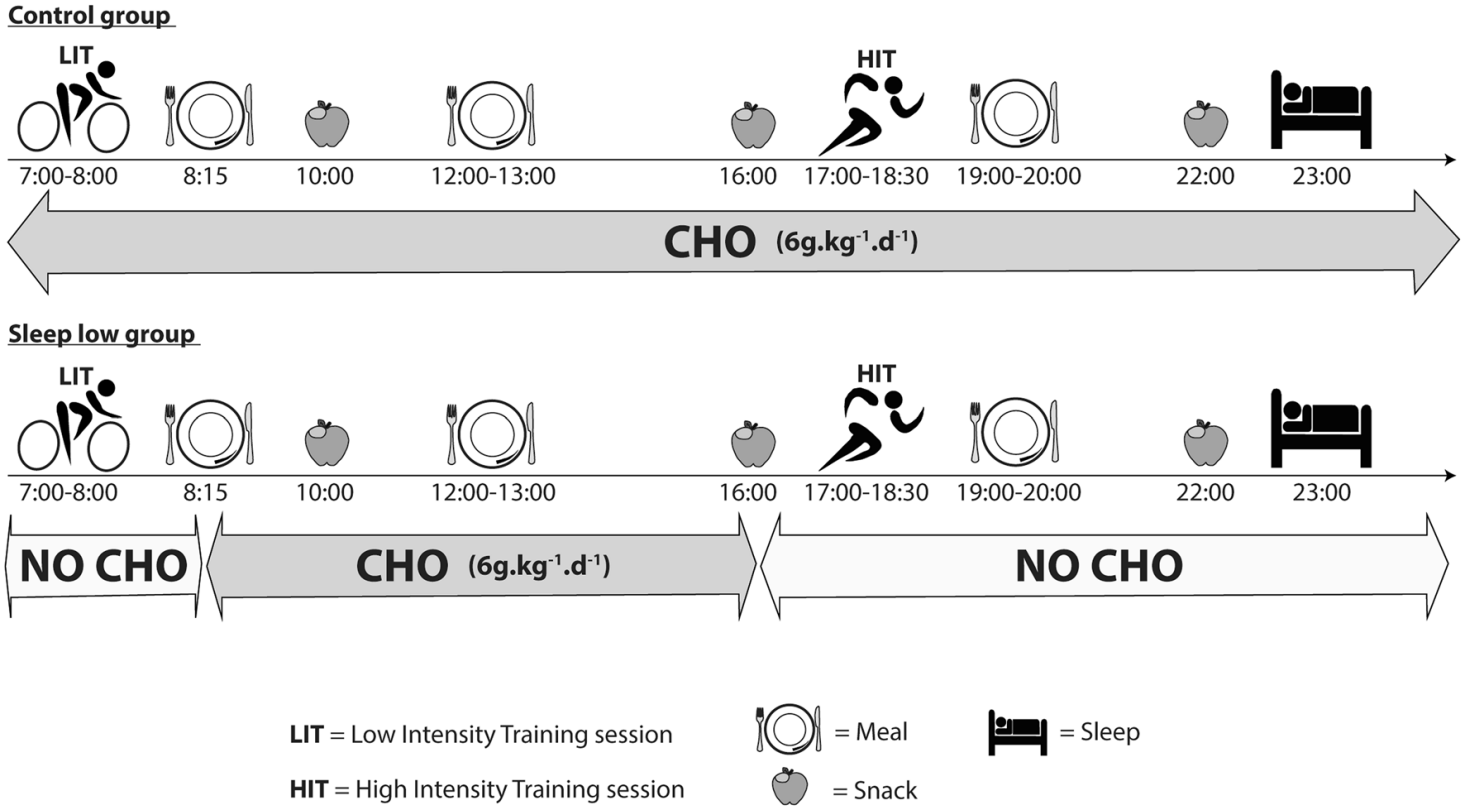
Daily CHO periodization for SL and CON groups during training days in phase II. CHO intake was distributed at every meal, snack and training sessions in the CON group, whereas it was concentrated only from breakfast (~8:15) to afternoon snack (~16:00) in the SL group

Before phase II, participants were randomly assigned to either the CON group (*n* = 10) or the SL group (*n* = 11) and had to follow different nutritional guidelines according to their group. In the SL group, no CHO intake was allowed for all HIT and LIT sessions. The dinner was also CHO-free and the LIT sessions were performed after an overnight fast so that they trained with low glycogen availability. On the contrary, the glycogen availability was regularly maintained in the CON group by consuming a sports drink (4.5 % CHO, Gatorade Performance Series^®^, Pepsico, USA) during training sessions and CHO at every meal. Finally all groups ingested the same amount of CHO per day (~6 g.kg^−1^.day^−1^) but allocated differently over each day. A typical daily CHO periodization for both groups during training days in phase II is depicted in Fig. 2. All participants received standardized dietary recommendation according to their membership group and their body weight. To avoid muscle catabolism, a high protein sugar-free drink (High Protein 15 g, 20 mL, UHS Bruno, France) was prescribed just before going to bed.

The participants had to fill in a food diary during the last week in phase I and phase II. They were instructed to fill in it the most detailed as possible (weighed food, pictures of dishes, details of the use of cooking fat, type and quantity of oil used for dressing…). The diaries were analyzed by the same scientist with the software Nutrilog 2.31 (Nutrilog SAS, France).

### Measurements

#### $\dot{V}{\text{O}}_{2\hbox{max} }$ and performance tests

For their first visit to the lab, subjects underwent an incremental cycling test at a self-selected cadence on an electronically braked cycle ergometer (Excalibur Sport, Lode^®^, Groningen, The Netherlands). The test consisted of a warm-up lasting 6 min at 100 W followed by an incremental period in which power output was increased by 25 W every 2 min until volitional exhaustion. The test was performed until exhaustion to assess maximal oxygen uptake ($\dot{V}{\text{O}}_{2\hbox{max} }$) and maximal aerobic power (MAP). During the test, oxygen uptake ($\dot{V}{\text{O}}_{2}$), carbon dioxide uptake ($\dot{V}{\text{CO}}_{2}$), minute ventilation ($\dot{V}E$) and respiratory exchanges ratio (RER) were continuously recorded and monitored as breath by breath values (Quark, Cosmed^®^, Rome, Italy). The gas and flow analyzers were calibrated prior to each test using ambient air, known-concentration gas and a 3 L syringe. $\dot{V}{\text{O}}_{2\hbox{max} }$ was determined by the highest 30 s average value. MAP (*W*) was calculated as MAP = *W* completed + 25 × (*t*/120) where *W* is the last completed workload and t is the number of seconds in the last workload completed or not.

The performance test was the same presented in the recent study published by Marquet et al. (2016). Briefly it was organized to assess the potential changes in endurance performance in ecological condition. It was planned during the first week as a familiarization trial, and immediately before and after the Phase II. This test was designed to simulate the end of a triathlon race. The test started by 40 min cycling at 70 % MAP at a self-selected cadence, immediately followed by a 10 km simulated running race. To allow the subject to drink during the exercise, two short active rest periods (30 s at 100 W at minutes 15 and 30) were organized, during which a water bottle was given to the subject. Immediately after the cycling exercise, the subjects quickly moved to the running track (340 m indoor) to start a 10 km test. During this test, subjects did not wear any apparatus and could drink a CHO-rich drink (4.5 g CHO per liter, Gatorade Performance Series-Endurance Formula) whenever they wanted. The bottle was placed on a table positioned on the running track. The bottle was regularly replaced on the table after each drink and weighed before and after the running test to evaluate the fluid intake. No significant difference was observed for the quantity of CHO ingested between performance tests (*P* = 0.62) and between groups (respectively for SL group; PRE vs. POST 14.7 ± 7.21 vs. 15.3 ± 6.43 g; *P* = 0.47 and for CON group PRE vs. POST 18.0 ± 15.5 vs. 15.1 ± 11.3 g; *P* = 0.50) The time lap was continuously recorded by an experimenter positioned on the track.

#### Blood collection and analyses

To avoid interassay variation, all blood samples were analyzed in a single batch at the end of the study. In five occasions (before and after the phase II, and before the last training session each week of the phase II, day 4, 11 and 18) in a fasted state, blood samples were collected from a superficial forearm vein using standard venipuncture techniques. 33 mL of blood was directly collected into EDTA tubes (2 EDTA tubes = 6 mL and 1 EDTA tube = 3 mL) for each sample (Greiner Bio-one; Frickenhausen, Germany).

Blood samples were immediately centrifuged at 4000 rev min^−1^ for 10 min at +4 °C to separate plasma from red blood cells. The obtained plasma sample was then stored in multiple aliquots (Eppendorf type, 1500 μL per sample) at −80 °C until analysis. From these samples, cortisol and vitamin D concentrations were determined in plasma with commercially available high sensitivity ELISA kits (R&D Systems, Minneapolis, MN, USA). The assay for [cortisol]b had an intraassay CV of 9.2–6.3 % over a concentration range of 1.3–6.5 µg L^−1^ and an interassay CV of 21.2–10.4 % over 1.1–5.5 µg L^−1^. The assay for [vitamin D]b had an intraassay CV of 5.7–6.2 % over a concentration range of 33–180 ng mL^−1^ and an interassay CV of 5.1–7.4 % over 52.9–164 ng mL^−1^. All blood samples were analyzed in duplicate at respective wavelength on a spectrophotometer Dynex MRXe (Magellan Biosciences, Chelmsford, MA, USA). Blood from 3 mL tubes was analyzed for leukocyte count using an automated cell counter (Cell-DynH RubyTM, Abbott, IL, USA) and standard laboratory procedures.

#### Saliva collection and analyses

On five occasions (before and after the phase II, and before the last training session each week of the phase II, day 4, 11 and 18) in a fasted state, athletes provided saliva samples. The samples were collected in multiple sterile aliquots (Eppendorf type, 3000 μL per sample) over a timed 5-min period and stored at −80 °C until assay. After thawing, saliva samples were centrifuged at 4000 rev min^−1^ for 10 min at +4 °C. The samples were analyzed for salivary immunoglobulin-A (sIgA) and interleukin-6 (IL-6) in accordance with the manufacturer’s recommendations (EIA kits, Salimetrics©, State College, PA, USA). The assay for [IgA]s had an intraassay CV of 4.5–6.9 % over a concentration range of 91.1–805.4 µg mL^−1^ and an interassay CV of 8.9–8.6 % over 25.3–204.1 µg mL^−1^. The assay for [IL-6]s had an intraassay CV of 3–10 % over a concentration range of 4–323 pg mL^−1^ and an interassay CV of 8–6 % over 9–342 µg mL^−1.^

#### Illness symptoms

The occurrence of upper tract respiratory infections (URTI) was evaluated using the Wisconsin Upper Respiratory Symptom Survey-21 (WURSS-21) (Barrett et al. 2009). The WURSS-21 includes one global severity question, ten symptom-based questions, nine functional impairment or quality-of-life questions, and one global change question. The severity of each reported symptom was rated on a seven-point scale: 1 (very mild), 3 (mild), 5 (moderate), and 7 (severe). An overall symptom score was calculated by adding the severity scores from all items except the first and the last as they have categorically different reference domains. The questionnaire was performed every day on the 3 weeks of phase II, and was administered at the same period of the day in a quiet place. All athletes had prior knowledge about the completion of the questionnaire. For comparison between weeks, the average of all answered questionnaires at each stage was used. Higher scores indicate more severe symptoms (the theoretical maximum score being 133) whilst a score of 0 indicates the complete absence of symptoms.

#### Sleep monitoring

During both phases I and II, subjects’ sleep patterns (i.e., sleep quantity and quality) were monitored continuously using an Actiwatch worn on the non-dominant wrist (Cambridge Neurotechnology Ltd., UK) with the epoch length set to 1 min. Athletes were monitored in their home environment every day during phase I (21 days), and during phase II (21 days). Mean behavioral activity over the entire recording period was automatically calculated using the Sleepwatch software (Actiwatch activity and sleep analysis version 5.28, Cambridge Neurotechnology, Ltd.). Wristwatch actigraphy is a non-intrusive tool used to estimate sleep efficiency, which has been validated for reliability (Sadeh 2011). When compared with polysomnography, results show an accuracy of up to 80 % in sleep disordered patients for total sleep time and sleep efficiency (Kushida et al. 2001).

Sleep-wake scoring can be reliably obtained only with additional information provided by manually completed sleep logs (Fietze et al. 2009). All participants were, therefore, requested to complete daily sleep diaries, indicating the times of going to bed, falling asleep, waking up, and leaving the bed. In addition, participants were asked to mark the time of switching off the light to sleep and wake-up time by pressing the button on the face of the Actiwatch.

Individual nights of sleep were analyzed for the following range of variables:

- time in bed (h): the total amount of time spent in bed between bedtime and get-up time;
- bedtime (hh:mm): the self-reported clock time at which a participant went to bed to attempt to sleep;
- get-up time (hh:mm): the self-reported clock time at which a participant got out of bed;
- sleep latency (min): the amount of time between bedtime and sleep start;
- actual sleep time (hh:mm): assumed sleep time as determined by the algorithm, taking into account immobile time;
- sleep efficiency (%): actual sleep time expressed as a percentage of time in bed;
- fragmentation index: a measure of restlessness during sleep, using the percentage of epochs where activity is >0;
- immobile time (min): the actual time spent immobile in bed.

To quantify how the training weeks affected the perceived sleep quality, the participants reported their perceived feelings on a seven-point scale, going from *very, very good* (=1) to *very, very poor* (=7) after waking up each morning (Hooper et al. 1995).

### Statistical analysis

All statistical analyses were conducted using the software Statistica 6.1 (StatSoft). All data are expressed as mean ± SD. Normality of data was tested using a Shapiro–Wilk test. Values at baseline for age, body composition, and experience in endurance sport, MAP, *V*O_2max_ and dietary habits were compared between groups (i.e., sleep low, SL and sleep normal, CON) using a one-way ANOVA. Two-ways (group × time) ANOVA were used to examine differences in dependent variables (i.e., sleep characteristics, perceived sleep, illness symptoms, blood and saliva markers of immune response) between groups means at each time point of the protocol. When a significant main effect was found, pairwise comparisons were conducted using Newman–Keuls post hoc analysis. Effect sizes were also calculated using partial eta squared ($\eta_{\text{p}}^{2}$) values. Values of 0.1, 0.3 and over 0.5 were, respectively, considered as small, medium and large effects. For all tests, the significance level was set at *P* < 0.05.

## Results

Data presented in this article derived from the same experimental protocol presented by Marquet et al. (2016) and thus are complementary to those already presented by Marquet et al. (2016).

### Effects on chronometric performance on the 10 km running race

A significant enhancement of the chronometric performance on the simulated 10 km running race was recorded at the end of the training program for all participants of the SL group, whereas no difference was recorded in the CON group (for more details about performance tests, refer to Marquet et al. 2016).

### Effects on dietary patterns over the experimental protocol

The macronutrient intake significantly changed between phase II and phase I in similar proportions between SL and CON groups, mainly with an increase in carbohydrate and protein intake between phase I and II without significant changes in energy intake (for more details about the macronutrient intake, refer to Marquet et al. 2016).

As depicted in Table 1(A) and (B), the micronutrient intake (vitamin A, B_1_, B_2_, B_3_, B_6_, B_9_, B_12_, C, D, E and magnesium, calcium, phosphorus, potassium, sodium, iron, zinc, copper, manganese, selenium) was not significantly altered between phase II and phase I and no significant difference was recorded between SL and CON groups.

**Table 1:** (A) Vitamin and (B) minerals intake for SL and CON groups in phase I and phase II

	Phase I	Phase II
(A)		
Vit A (µg)		
SL	428.5 ± 238.8	281.8 ± 115.9
CON	367.9 ± 134.0	350.6 ± 101.8
Vit B_1_ (mg)		
SL	1.3 ± 0.5	1.2 ± 0.6
CON	1.4 ± 0.5	2.0 ± 0.7
Vit B_2_ (mg)		
SL	1.9 ± 0.6	1.6 ± 0.6
CON	2.0 ± 0.7	1.8 ± 0.4
Vit B_3_ (mg)		
SL	21.2 ± 6.2	25.4 ± 11.0
CON	25.3 ± 8.1	25.5 ± 8.9
Vit B_6_ (mg)		
SL	1.8 ± 0.4	2.0 ± 0.7
CON	2.1 ± 0.9	1.9 ± 0.5
Vit B_9_ (µg)		
SL	267.2 ± 86.8	293.9 ± 117.6
CON	309.4 ± 155.3	296.1 ± 81.4
Vit B_12_ (µg)		
SL	4.4 ± 2.0	4.4 ± 1.5
CON	4.4 ± 1.7	4.4 ± 0.4
Vit C (mg)		
SL	109.2 ± 50.4	130.7 ± 50.4
CON	141.0 ± 113.2	132.3 ± 37.9
Vit D (µg)		
SL	9.3 ± 2.2	9.3 ± 0.7
CON	9.4 ± 0.9	9.2 ± 1.1
Vit E (mg)		
SL	8.4 ± 3.2	7.4 ± 3.1
CON	7.3 ± 1.4	5.6 ± 0.9
(B)		
Magnesium (mg)		
SL	350.6 ± 110.5	383.7 ± 143.3
CON	406.7 ± 131.0	416.3 ± 171.1
Calcium (mg)		
SL	788.2 ± 315.8	844.5 ± 229.8
CON	961.0 ± 286.2	950.4 ± 215.6
Phosphorus (mg)		
SL	1242.9 ± 370.3	1485.8 ± 332.8
CON	1606.5 ± 540.9	1662.6 ± 371.0
Potassium (mg)		
SL	2896.4 ± 620.0	3176.0 ± 934.1
CON	3413.8 ± 1267.9	3269.9 ± 680.9
Sodium (mg)		
SL	5114.4 ± 2181.0	4908.5 ± 655.7
CON	4933.8 ± 1064.2	5599.0 ± 1057.2
Iron (mg)		
SL	12.1 ± 2.7	12.2 ± 3.8
CON	11.7 ± 5.2	11.1 ± 2.1
Zinc (mg)		
SL	9.1 ± 3.2	10.0 ± 2.6
CON	10.1 ± 3.6	10.8 ± 2.1
Copper (mg)		
SL	1.4 ± 0.6	2.1 ± 1.9
CON	1.5 ± 0.6	1.5 ± 0.2
Manganese (mg)		
SL	3.2 ± 1.5	3.8 ± 1.4
CON	3.0 ± 1.3	3.6 ± 0.9
Selenium (µg)		
SL	56.7 ± 17.6	53.3 ± 21.5
CON	52.9 ± 17.6	56.3 ± 25.8

Data are mean ± SD

### Effects on blood and saliva immune and inflammatory variables

**Fig. 3:**
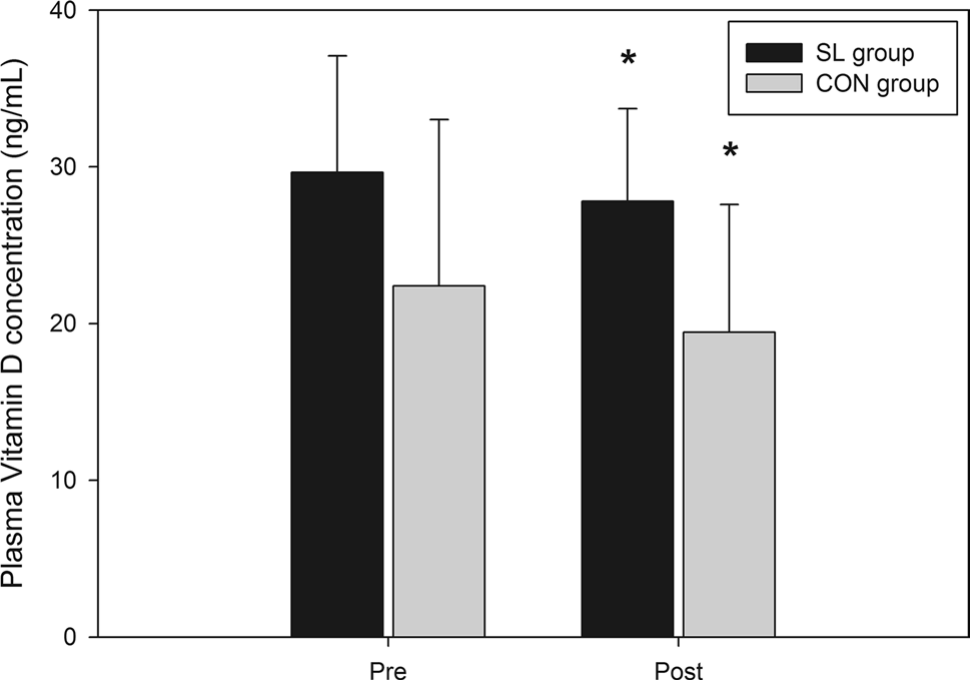
Evolution of plasma vitamin D concentration for SL and CON groups from pre to post phase II. *Significantly different from pre values (*P* < 0.05)

There were no significant differences in circulating numbers of leukocytes, neutrophils, monocytes, eosinophils, basophils or lymphocytes between groups and between the different phases of the protocol (Table 2). Plasma cortisol levels were not modified throughout the protocol and were not different between groups (Table 3). No significant change was recorded in salivary IL-6 concentration (Table 3). Salivary IgA decreased over the experimental protocol only in the SL group (Table 3; from 391.8 to 245.1 µg L^−1^, *P* < 0.05, *d* = 0.23 from PRE to POST intervention). The vitamin D status decreased along the protocol in similar proportions between groups (Fig. 3; from 29.6 ± 7.4 to 27.8 ± 5.9 ng mL^−1^ and from 22.4 ± 10.6 to 19.4 ± 8.1 ng mL^−1^, *P* < 0.05, *d* = 0.18 between PRE and POST intervention, in SL and CON groups, respectively).

**Table 2:** Weekly evolution of plasma white blood cell counts for SL and CON groups from pre to post phase II

	Pre	Phase II			Post
		Week 1	Week 2	Week 3	
Leucocytes (/mm^3^)					
SL	4981.8 ± 1697.5	5000.0 ± 1649.8	5118.2 ± 1462.1	4800.0 ± 1485.5	5230.0 ± 1682.6
CON	5870.0 ± 1110.6	5900.0 ± 829.2	5450.0 ± 1095.7	5387.5 ± 800.8	5188.9 ± 871.0
Neutrophils (/mm^3^)					
SL	2695.6 ± 1442.2	2590.6 ± 1000.5	2781.1 ± 1047.9	2541.9 ± 1127.9	3044.7 ± 1473.4
CON	3109.2 ± 1079.2	3211.2 ± 539.9	2795.8 ± 867.6	2699.1 ± 545.4	2512.6 ± 681.5
Eosinophils (/mm^3^)					
SL	111.7 ± 58.0	101.0 ± 57.7	107.6 ± 65.4	94.7 ± 63.8	111.8 ± 61.3
CON	172.0 ± 97.8	172.9 ± 83.6	166.8 ± 108.5	170.9 ± 84.9	150.7 ± 96.5
Basophils (/mm^3^)					
SL	28.5 ± 14.9	24.7 ± 17.7	39.8 ± 16.9	34.7 ± 16.4	39.6 ± 24.7
CON	25.3 ± 9.0	28.4 ± 5.4	22.6 ± 13.5	25.9 ± 12.5	35.7 ± 28.7
Lymphocytes (/mm^3^)					
SL	1736.4 ± 411.1	1907.5 ± 826.7	1697.4 ± 444.3	1737.3 ± 498.9	1634.2 ± 456.1
CON	2088.9 ± 543.5	2011.9 ± 542.2	1993.5 ± 480.7	2013.9 ± 520.1	2013.4 ± 743.3
Monocytes (/mm^3^)					
SL	409.8 ± 168.9	376.5 ± 152.0	492.1 ± 331.1	391.5 ± 133.4	399.9 ± 156.0
CON	475.0 ± 180.0	475.8 ± 115.1	471.9 ± 99.2	478.8 ± 104.7	477.0 ± 117.6

Data are mean ± SD

**Table 3:** Weekly evolution of salivary IgA and IL-6 concentrations and plasma cortisol concentration for SL and CON groups from pre to post phase II

	Pre	Phase II			Post
		Week 1	Week 2	Week 3	
sIgA (µg/mL)					
SL	353.7 ± 188.7	319.1 ± 167.5	286.7 ± 154.5	297.4 ± 132.5	241.6 ± 98.1*
CON	390.6 ± 235.0	270.5 ± 139.7	359.2 ± 148.4	382.6 ± 168.5	348.3 ± 153.9
sIL-6 (pg/mL)					
SL	2.6 ± 0.3	2.6 ± 0.4	2.7 ± 0.4	2.6 ± 0.4	2.6 ± 0.5
CON	2.8 ± 0.3	2.8 ± 0.2	2.6 ± 0.4	2.7 ± 0.4	2.7 ± 0.5
Cortisol (µg/L)					
SL	157.2 ± 40.2	140.4 ± 34.9	159.3 ± 26.2	155.6 ± 28.8	148.0 ± 34.0
CON	177.1 ± 30.1	180.6 ± 38.9	169.9 ± 39.3	176.2 ± 36.7	155.7 ± 33.7

Data are mean ± SD

* Significantly different from values recorded in pre (*P* < 0.05)

### Effects on the incidence of URTI

**Fig. 4:**
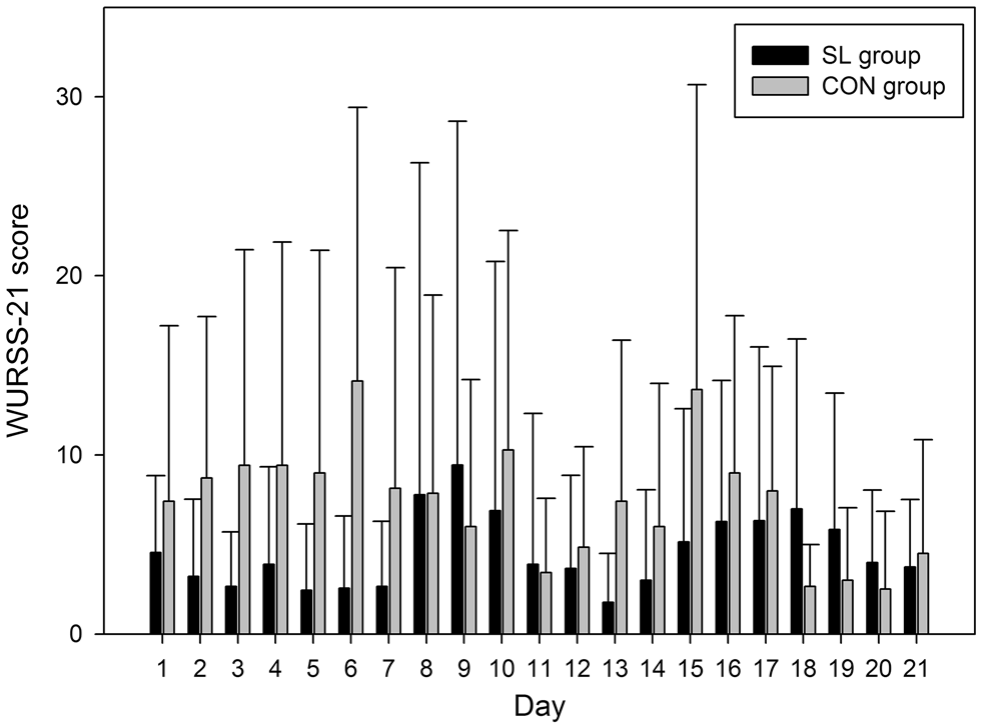
Daily evolution of the WURSS-21 scores for SL and CON groups during the 21 days of diet and training intervention in phase II

The distribution of the daily WURSS-21 scores for both groups is depicted in Fig. 4. The 3 weeks of sleep low program did not modify significantly the WURSS-21 scores, in comparison with the CON group. The SL group’s average score (4.5) was very low and not found to differ to a significant extent compared to the CON group (7.7). The maximum scores recorded were 59 for one subject (1 day in the second week) of the SL group and 39 for one subject in the CON group (1 day in the first week).

### Effects on perceived sleep quality and sleep actigraphy

**Fig. 5:**
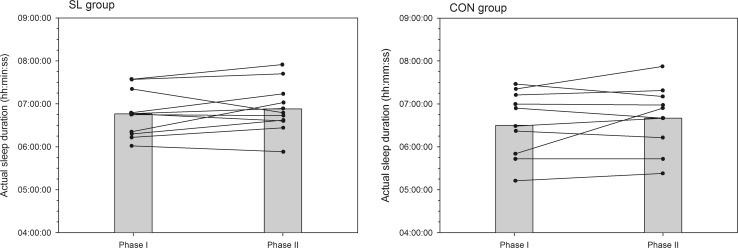
Mean (*shaded bars*) and individual cases of actual sleep duration for SL and CON groups during phase I and phase II

**Fig. 6:**
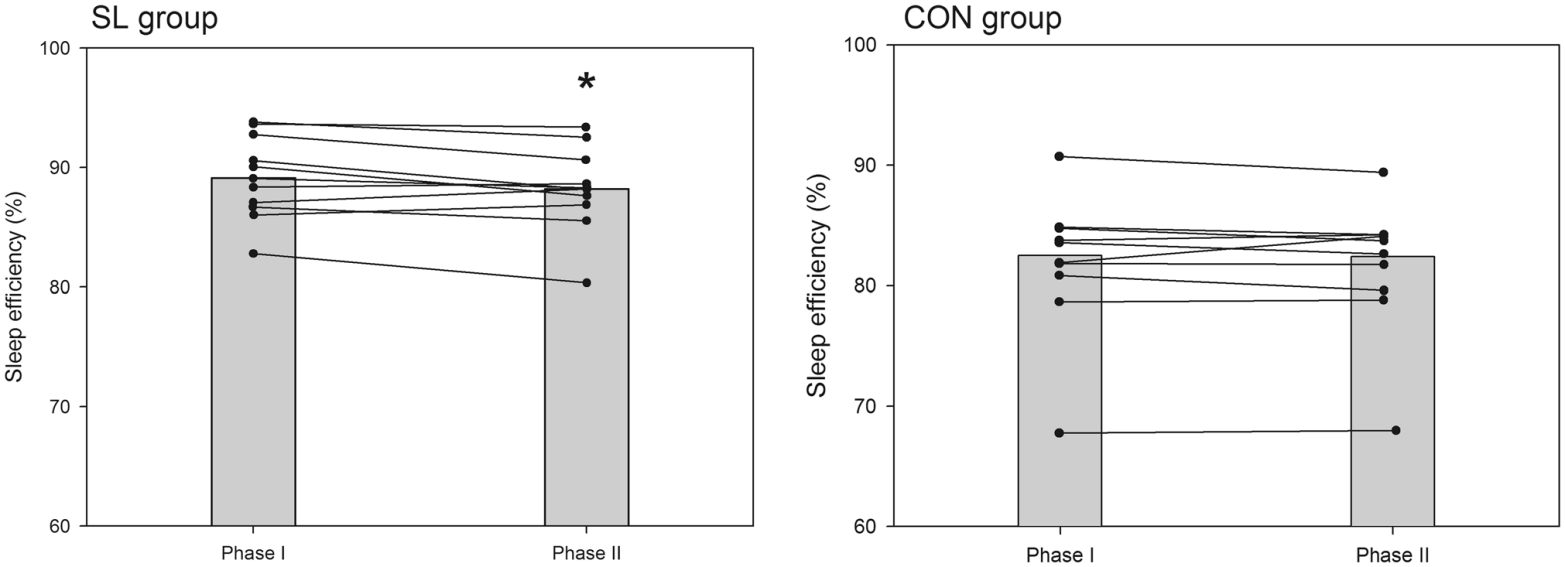
Mean (*shaded bars*) and individual cases of sleep efficiency for SL and CON groups during phase I and phase II. *Significantly different from values recorded in phase I (*P* < 0.05)

**Fig. 7:**
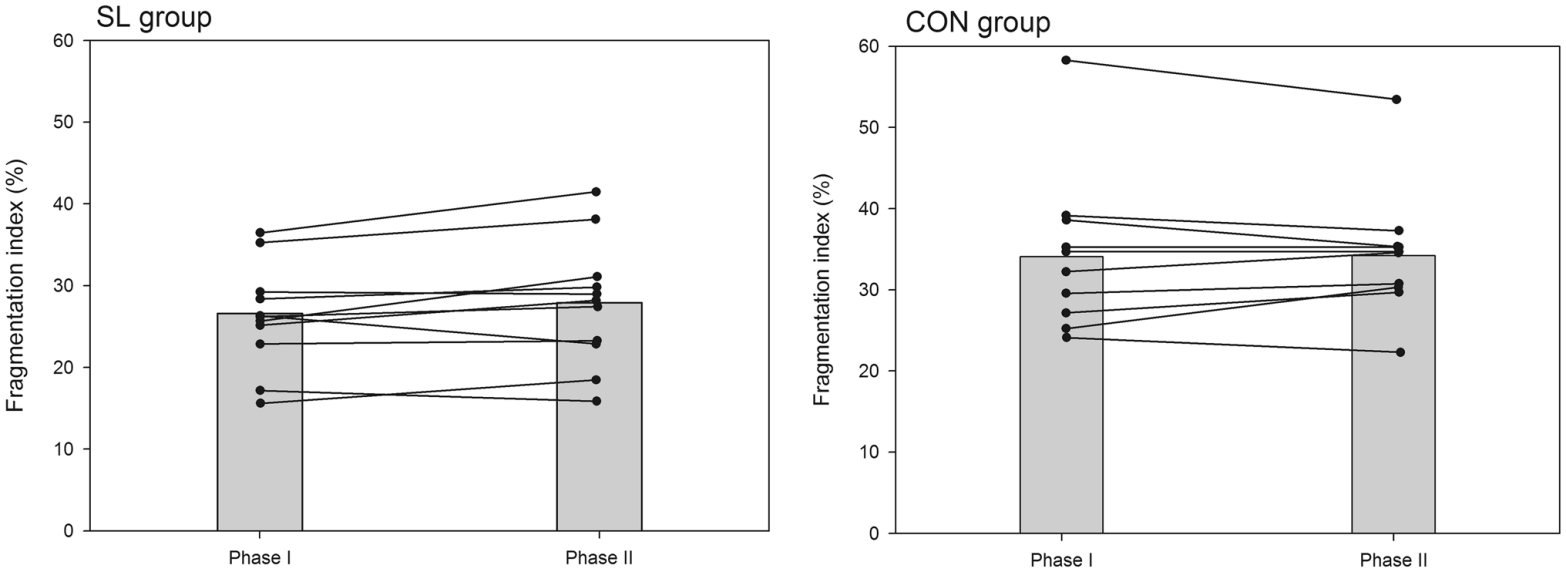
Mean (*shaded bars*) and individual cases of fragmentation index for SL and CON groups during phase I and phase II

A summary of variables related to sleep quality and quantity is presented in Table 4. There was no significant time-group interaction in perceived sleep quality, get-up time, sleep latency, fragmentation index, and number of immobile minutes between phases I and II. In both groups, all subjects went to bed earlier (on average −20 and −27 min in the SL and CON groups, respectively, *P* < 0.05, *d* = 0.28) in the phase II than phase I and woke-up at the same time. As such, time in bed significantly increased in similar proportions in both groups (on average +13 and +15 min in the SL and CON groups, respectively, *P* < 0.05, *d* = 0.22). However, as depicted in Fig. 5, the actual sleep duration (i.e., the time asleep from sleep start to sleep end, less awakening episodes) was not significantly modified between phase I and II in both groups. Sleep efficiency slightly decreased only in the SL group (Fig. 6; *P* < 0.05, *d* = 0.25) and the fragmentation index tended to increase only in the SL group (Fig. 7; *P* = 0.06).

**Table 4:** Evolution of sleep actigraphy data for SL and CON groups between phase I and phase II

	Phase I	Phase II
Perceived sleep quality (AU)		
SL	3.1 ± 0.5	3.1 ± 0.7
CON	3.4 ± 0.4	3.4 ± 0.3
Time in bed (h:min)		
SL	7:35 ± 0:24	7:48 ± 0:31*
CON	7:48 ± 1:00	8:03 ± 0:45*
Bed time (hh:min)		
SL	23:53 ± 0:37	23:33 ± 0:40*
CON	23:46 ± 1:08	23:19 ± 0:38*
Get-up time (hh:min)		
SL	7:28 ± 0:41	7:23 ± 0:43
CON	7:45 ± 0:59	7:39 ± 0:48
Sleep latency (min)		
SL	6 ± 6	8 ± 4
CON	10 ± 7	11 ± 8
Actual sleep time (h:min)		
SL	6:46 ± 0:32	6:53 ± 0:36
CON	6:30 ± 0:50	6:40 ± 0:47
Sleep efficiency (%)		
SL	89.1 ± 3.6	88.2 ± 3.7*
CON	82.5 ± 6.8	82.4 ± 5.9
Fragmentation index (%)		
SL	26.6 ± 6.7	27.9 ± 8.3
CON	34.1 ± 11.3	34.2 ± 8.6
Immobile minutes		
SL	392 ± 31	399 ± 34
CON	386 ± 51	396 ± 44

Data are mean ± SD

* Significantly different from values recorded in phase I (*P* < 0.05)

## Discussion

A growing number of studies support the interest of performing some training sessions with a low glycogen availability to enhance the adaptation to training and endurance performance. In a recently published work from our team (Marquet et al. 2016), we showed a significant improvement in endurance performance and running efficiency following a 3-week training program including the sleep low strategy, i.e., delaying the replenishment of glycogen stores over night and training fasted the next morning. However, reducing the energy availability around training sessions is also well known to increase the risk of mal adaptation to training, including immunodepression and an increased incidence of URTI. This is the reason why we also investigated the effect of the sleep low training program presented by Marquet et al. (2016) on selected immune parameters, the prevalence of URTI and sleep disturbances in endurance athletes. One of the main complementary findings presented in this article is that the enhancement of performance previously described after the SL protocol (Marquet et al. 2016) was accompanied by a slight decrease in sleep efficiency and slight alterations of some indicators of immune function.

### Effects on immune parameters and the incidence or URTI

The effects of exercise on immune function are well documented in the literature. Taken together, results indicate that exercise can have either positive or negative impact on immunity, depending on the nature, intensity and duration of exercise, as well as athlete fitness (Nieman 2000). Regular moderate exercise may enhance immunity and lower the risk or URTI by 20–45 % compared with a sedentary lifestyle (Matthews et al. 2002; Nieman et al. 2011). On the other hand, heavy exercise or periods of chronic exercise may impair immune function and raise the risk of URTI by decreasing resting levels of saliva secretory immunoglobulin-A (sIgA), leukocytes and neutrophil function (Fahlman and Engels 2005; Gleeson 2007; Gleeson et al. 1999). In addition, inadequate dietary feedings such as deficiency in specific macro- and/or micronutrients or negative energy balance, may contribute to impaired immunity and increase the risk of infection (Gunzer et al. 2012). Interestingly, in this study, resting immune variables were not significantly modified by the training/nutrition intervention, and the incidence of URTI was not increased in both groups. The number of white blood cells and the incidence of URTI were not significantly altered by the 3 weeks of training, comprising yet nine high intensity training (HIT) sessions and nine low intensity training (LIT) sessions performed after an overnight fast. This result was unexpected considering the high risk of immunodepression classically reported during the early recovery period after HIT sessions, and accentuated by the deprivation in carbohydrates (Gunzer et al. 2012). Only the vitamin D status decreased from pre to post intervention but in similar proportions between groups. This decrease in vitamin D was accompanied with a slight decrease in sIgA concentration (*d* = 0.23) only in the SL group over weeks of training. This result is in conformity with previous studies reporting a positive correlation between the vitamin D status and the sIgA secretion (He et al. 2014). However, it is worth noting that the decrease in vitamin D could have been also induced by the low direct exposition to UVB, since this study was conducted in the winter season at a high latitude (Paris, 53°N). While the mean vitamin D was quite low for all participants, no athletes were deficient in vitamin D at the end of the protocol.

In our study, the absence of marked perturbations of immunity and URTI despite a chronic physiological stress could be explained by the particularity of our nutrition guidelines. Indeed, participants of the SL group were asked to perform all HIT sessions with high glycogen availability, whereas LIT sessions were performed with low glycogen availability after an overnight fast. However, the total daily carbohydrate intake (5.44 ± 1.20 g kg^−1^) and energy intake (2684 ± 500 kcal) were maintained similar to those of the CON group (5.65 ± 0.99 g kg^−1^ and 2837 ± 505 kcal), likely allowing the maintenance of the immune function throughout the protocol (Marquet et al. 2016). Indeed, carbohydrates are an important source of energy for immune cells (including lymphocytes, neutrophils and macrophages) because their metabolic rates are extremely high (Gunzer et al. 2012). Most importantly all HIT sessions were performed with high glycogen stores, likely inducing a favorable effect on immunity. Costa et al. (2005) reported that training with high CHO availability lead to a stable glucose level, decreased plasma cortisol level, and an increase in sIgA during 1 week training in well trained triathletes. On the contrary, maintaining a low CHO diet for the entire week induced a significant increase in cortisol level. In our study, cortisol level was not altered by the nutrition manipulation, probably explaining in large part the maintenance of immune function for all our participants. Indeed, cortisol is known to have a suppressive effects on leukocyte function including immunoglobulin production, lymphocyte proliferation and NK cell activity (Bishop et al. 1999). CHO availability may also increase the anti-inflammatory cytokine response to exercise, as shown by Bishop et al. (2001b) through a significant increase in IL-6 concentration further to a cycling exercise performed in a low CHO diet (<1 g kg^−1^ per day) for 3 days prior to the exercise. On the contrary, in our study, IL-6 concentration was not significantly modified, probably explained by the CHO deprivation occurring only during night hours in each day. Finally one other factor which could explain the absence of immunosuppression in the SL group was the intake of a protein snack (15 g protein, 0 g CHO) before going to bed. Initially provided to limit the potential protein catabolism process during the night, ingesting protein in the recovery phase after HIT sessions might have contributed to the maintenance of immune function. Among protein ingested, the specific role of the glutamine amino acid as a privileged energy provider to lymphocytes, macrophages and neutrophils, might be hypothesized (Hiscock and Pedersen 2002). Hence, ingesting a protein shake after exercise might have compensated the fall in plasma glutamine and associated immunosuppression. Moreover, in the SL group the CHO deprivation period occurred only during night— period during which participants slept and were not exposed to potential pathogen elements and stressors to the immune system such as cold, other people, or mental stress. Finally, well-maintained sleeping patterns throughout the protocol might likely have contributed to the absence of profound perturbations of the immune system and on the very low incidence of URTI.

### Effects on sleeping patterns

Since the first studies having reported that a lack of sleep may significantly alter metabolic, immune and cognitive function, the analyses of sleeping patterns in the sporting context have increased massively (Fullagar et al. 2015). Signs of sleep disturbances (e.g., troubles to fall asleep, increase of wake periods) are often recorded in particular after competitions or high intensity training sessions (Fullagar et al. 2015). For example, Hausswirth et al. (2014) have reported a significant decrease in sleep quality and quantity in triathletes involved in a 3-week overload training program (i.e., +30 % of habitual training load). In this latter study, the actual sleep duration was decreased by ~30 min every night during the training program, accompanied by a reduction of sleep efficiency and immobile time. Sleep disruptions are also classically reported in athletic populations training temporarily at high altitude or in hot conditions during training camps (Buchheit et al. 2016; Sargent et al. 2013). Taken together, all data from the literature suggest that intensified training and/or the exposition to physiological stressors (i.e., heat, altitude) increase the risk of sleep disturbance. Within this framework, it can be hypothesized that increasing the physiological stress through a nutritional manipulation during the recovery period could increase and even accentuate the risk of sleep disturbances.

To the best of our knowledge, this study is the first to provide an objective report of sleeping patterns in two groups of trained athletes under different nutrition guidelines (i.e., sleep with low glycogen availability vs. sleep with normal glycogen availability). This experimental situation involved a profound change of dietary habits for all participants, potentially increasing the risk of sleep disturbances. Indeed, although carbohydrates are generally recommended at night to facilitate falling asleep (Halson 2014), in our study they were avoided and replaced only by large amounts of vegetables and protein. However, surprisingly sleeping patterns were not markedly modified by the training/nutritional intervention. Sleep latency was not altered and participants even went to bed earlier (−13 and −15 min, *P* < 0.05, in the SL and CON group, respectively), while get-up time was not modified. In consequence, actual sleep duration tended to be greater in both groups (+7 and +10 min, NS, in the SL and CON group, respectively). These results suggest that spontaneously, participants went to bed earlier probably because of the fatigue felt after evening training sessions. The sleep quality was slightly altered since sleep efficiency decreased in the SL group (−1.1 %, *d* = 0.25) and the fragmentation index tended to increase (+4.1 %, *P* = 0.06), showing a small increase in wake episodes overnight. However, the overall perceived quality of sleep was not altered throughout the protocol suggesting only minor changes in sleep quality. Altogether, our results suggest that maintaining a normal daily CHO and calorie intake was effective in maintaining normal sleeping pattern, allowing a good adaptation to training.

### Implications for the manipulation of glycogen availability in training programs

This study has practical implications regarding the withholding of carbohydrates and lowered glycogen availability implemented during short-term endurance training programs: it reveals a minimal effect on immune response and no effects on the occurrence of URTI; it reveals a minimal effect on sleeping patterns in trained triathletes; it raises new insight on the distribution of carbohydrates around training sessions to avoid maladaptation.

Although manipulating carbohydrate availability around training sessions is more and more used by endurance athletes to accentuate the training stimulus and get greater adaptation, to date no study had analyzed the potential side effects on the immune function. As inferred through objective markers of systemic and mucosal immune function as well as subjective markers of infection, the sleep low strategy (3 nights per week over 3 weeks) had no deleterious impact on the immune function. This positive result is likely explained by the maintenance of normal daily CHO (around 6 g kg^−1^ day^−1^) and energy intake (i.e., the CHO intake was matched between groups in this study). However, additional studies are necessary to confirm this result and analyze the impact of various dietary/training manipulations on the immune function. Moreover, in our study, the vitamin D status decreased in both groups accompanied with a slight reduction in the sIgA secretion in the SL group (*d* = 0.23), suggesting a slight reduction in immune defense. Accordingly, a preliminary vitamin D status assessment possibly followed by a dietary supplementation in vitamin D should be considered to optimize the immune status before engaging in a sleep low program, especially if started in the winter season.

Sleeping is often considered by coaches and trainers as the most effective recovery strategy. However, very limited data is available on the potential factors of influence, such as dietary feedings according to the distribution of training sessions. This study shows for the first time that sleeping patterns of trained athletes were not altered by a sleep low training strategy. Maintaining a normal CHO and energy intake every day of the training program would be enough to counterbalance the potential negative impact of withholding CHO in the recovery phase after exercise before going to bed.

This study provides new evidence on the optimization of the carbohydrate distribution over day to limit the risk of maladaptation to training. High intensity training sessions should be performed with high glycogen availability to maintain blood glucose concentration and thus immune function. The deprivation in CHO should occur in the recovery phase after HIT sessions organized overnight, to facilitate the glycogen depletion. Indeed, the night can be considered as a suitable period to withhold CHO (more convenient and easiest to endure a 7–8 h period of low glycogen availability). Ingesting a dinner rich in vitamin, minerals (mainly from vegetables) and proteins might reinforce the immune defenses. Exercises with low glycogen availability should be performed at a low intensity (fasted in the morning) to lower the risk of infection. Finally, a particular attention should be brought on the ingestion of sufficient amounts of CHO between morning and evening sessions (from breakfast to afternoon snack) to provide a normal amount of CHO (around 6 g kg^−1^ day^−1^ in this study). The use of liquid sources of CHO like sports drinks and gels can facilitate the ingestion of enough CHO in a short period of time.

## Conclusion

The purpose of this study was to test the effects of a 3-week sleep low training strategy (involving a withholding of CHO overnight) on immune function, the incidence or URTI and sleep patterns in trained endurance athletes. While the CON group maintained a normal CHO availability all time during the protocol, the SL group stopped CHO intake from the afternoon training session until the next morning training session, so that they spend all night with reduced glycogen availability. The main findings were that: (i) markers of systemic and mucosal immunity and the incidence of URTI were not significantly modified by the dietary intervention; (ii) sleep efficiency slightly decreased and the fragmentation index tended to increase only in the SL group. Overall, results derived from this study suggest that the risk of maladaptation to training is minimal when withholding CHO overnight between high and low intensity training sessions, in the condition of maintaining a normal daily CHO and energy intake concentrated earlier over day.

## Abbreviations

ANOVA: Analysis of variance
CHO: Carbohydrate
CON group: Control group
HIT: High intensity training
LIT: Low intensity training
MAP: Maximal aerobic power
RER: Rate of exchange ratio
sIgA: Salivary immunoglobulin-A
sIL-6: Salivary interleukin-6
SL group: Sleep low group
URTI: Upper respiratory tract infection
*V*CO_2_: Carbon dioxide uptake
*V*O_2_: Oxygen uptake
*V*O_2max_: Maximal oxygen uptake
WURSS-21: Wisconsin upper respiratory symptom survey-21
